# TrkAIII Promotes Microtubule Nucleation and Assembly at the Centrosome in SH-SY5Y Neuroblastoma Cells, Contributing to an Undifferentiated Anaplastic Phenotype

**DOI:** 10.1155/2013/740187

**Published:** 2013-06-06

**Authors:** Antonietta R. Farina, Natalia Di Ianni, Lucia Cappabianca, Pierdomenico Ruggeri, Marzia Ragone, Giulia Ianni, Alberto Gulino, Andrew R. Mackay

**Affiliations:** ^1^Department of Applied Clinical and Biotechnological Sciences, University of L'Aquila, Via Vetoio, Coppito 2, 67100 L'Aquila, Italy; ^2^Department of Experimental Medicine, University of Rome “La Sapienza,” 00185 Rome, Italy

## Abstract

The alternative TrkAIII splice variant is expressed by advanced stage human neuroblastomas (NBs) and exhibits oncogenic activity in NB models. In the present study, employing stable transfected cell lines and assays of indirect immunofluorescence, immunoprecipitation, Western blotting, microtubule regrowth, tubulin kinase, and tubulin polymerisation, we report that TrkAIII binds **α**-tubulin and promotes MT nucleation and assembly at the centrosome. This effect depends upon spontaneous TrkAIII activity, TrkAIII localisation to the centrosome and pericentrosomal area, and the capacity of TrkAIII to bind, phosphorylate, and polymerise tubulin. We propose that this novel role for TrkAIII contributes to MT involvement in the promotion and maintenance of an undifferentiated anaplastic NB cell morphology by restricting and augmenting MT nucleation and assembly at the centrosomal MTOC.

## 1. Introduction

TrkAIII is a developmentally regulated alternative splice variant of the NGF receptor tropomyosin-related kinase TrkA that is expressed by advanced stage human neuroblastomas (NBs), characterised by exon 6-7 skipping and exon 9 omission, and exhibits oncogenic activity in NB models [1–5]. TrkAIII oncogenic activity depends upon omission of the extracellular D4 Ig-like domain and several N-glycosylation sites, encoded within exons 6-7, important for receptor cell surface expression and prevention of ligand-independent activation [6, 7]. As a consequence and in contrast to fully spliced cell surface TrkA, TrkAIII exhibits intracellular expression and spontaneous, ligand-independent activation that is restricted to interphase within the intracellular membrane compartment. This results in chronic signalling through IP3k/Akt but not Ras/MAPK and promotes a more aggressive proliferating, undifferentiated stress-resistant, angiogenic, and tumourigenic stem cell-like NB cell phenotype, which is in stark contrast to ligand-activated cell surface TrkA, which signals through IP3k/Akt and Ras/MAPK and promotes a less aggressive phenotype characterised by neuronal differentiation associated with the inhibition of proliferation [1, 5, 8–10].

Microtubules (MTs) are dynamic polymers of *α*- and *β*-tubulins that play a central role in cellular differentiation [11–14]. In undifferentiated cells, MTs nucleate and assemble at the centrosome MT organising centre (MTOC), forming arrays of relatively short MTs that radiate outwards from the centrosome [11–15]. In differentiated cells, MTs differ in MTOC usage and nucleate also at cytoplasmic and/or perinuclear/Golgi-associated extracentrosomal MTOCs, resulting in the formation of more diffuse cytoplasmic MT mats and coils, and during neuronal differentiation, the formation of long MTs organised at the cell periphery is required for neuritogenesis and axon genesis [11–21].

Tyrosine kinases have been implicated in the regulation of MT nucleation, assembly, and stability. Src family tyrosine kinases Fyn and Lyn phosphorylate tubulin and reorganise MTs during monocyte differentiation [15]; Fyn and Syk kinases regulate MT organisation in mast cells [22]; Src recruits *γ*-tubulin ring structures to the centrosome and promotes MT nucleation and assembly through MAPK/Erk [23, 24]; c-Fes binds and phosphorylates *α*-tubulin and promotes tubulin polymerisation during myeloid hematopoietic cell and neuron differentiations [25]; insulin receptor tyrosine kinase phosphorylates tubulin and promotes interaction between PI3k and *γ*-tubulin required for insulin-induced MT reorganisation [26, 27]; and JAK mediates growth hormone-induced MT assembly [28]. TrkA has also been implicated in MT reorganisation. The neurotrophins NGF and NT-3 activate cell surface TrkA, which responds by recruiting MTs to detergent-resistant cell surface lipid rafts and reorganising the assembly of MTs required for neuritogenesis, axon genesis, and growth cone formation, resulting in neuronal differentiation [29–31]. Tyrosine-phosphorylated TrkA colocalizes with *α*-tubulin positive MTs during mitosis [32], interacts with and modifies *α*-tubulin [33], and moves along MTs during retrograde transport via an interaction with dynein [34]. 

We have previously reported that tyrosine-phosphorylated TrkAIII binds *γ*-tubulin and localises to the centrosome in human SH-SY5Y NB cells [4]. Here, we report that TrkAIII interacts with *α*-tubulin and promotes tubulin polymerisation, contributing to MT involvement in promoting and maintaining a proliferating, undifferentiated, and anaplastic NB cell phenotype by restricting and augmenting MT nucleation and assembly at the interphase centrosome.

## 2. Materials and Methods

### 2.1. Cell Lines and Reagents

Empty pcDNA control, TrkAI, TrkAIII, and Y670/674/675F kinase dead mutant TrkAIII (kd-TrkAIII) stable transfected SH-SY5Y NB cell lines have been described previously [1, 3, 4]. All cell lines were grown in recommended medium (RPMI or DMEM), supplemented with appropriate antibiotics (Zeocin for stable transfectants, penicillin, and streptomycin) and 10% foetal calf serum. Nocodazole was purchased from Sigma-Aldrich (St. Louis, MO), and the pan Trk inhibitor CEP-701 [35] was kindly supplied by Cephalon Inc. (West Chester, PA). Rhodamine-conjugated *α*-tubulin, microtubule polymerisation buffers, and associated reagents were purchased from Cytoskeleton (Denver, CO). VectorMount mounting medium for immunofluorescence (IF) was purchased from Vector Laboratories (Burlingame, CA). Monoclonal *α*-tubulin, polyclonal anti-carboxyl terminal TrkA (C14), and monoclonal anti-phosphotyrosine (pY99) antibodies were purchased from Santa Cruz (Santa Cruz, CA). Polyclonal antibodies against *γ*-tubulin and TrkA phosphoY490 were from Sigma-Aldrich (St. Louis, MO). FITC and Texas red-conjugated secondary anti-mouse and anti-rabbit IgG antibodies were from Jackson Immune Research (Bar Harbor, Maine).

### 2.2. Indirect IF

Cells grown on Nunc glass chamber slides (Sigma-Aldrich) were washed in PBS, fixed in 96% ethanol-3% glacial acetic acid, and processed for indirect IF. Fixed slides were incubated for 1 h in blocking solution (1% bovine serum albumin in PBS-0.03% Triton X-100) and then for 2 to 16 h with primary antibody in blocking solution at room temperature. Slides were then washed three times in PBS-0.03% Triton X-100, incubated with secondary fluorochrome-conjugated antibody diluted in blocking solution for 1 hour at room temperature, washed in PBS-0.03% Triton X-100, and mounted using VectorMount. IF images were obtained using a Zeiss “Axioplan-2” fluorescence microscope, fitted with a digital camera, and images were processed using Leica M500 Image Manager software.

Nuclear lobulation was studied by fluorescent DAPI (Vector Labs) staining of nuclear chromatin.

### 2.3. Microtubule Regrowth Assay

Microtubule regrowth assays were performed as previously described [23]. Briefly, subconfluent (80%) cell cultures grown on Nunc glass chamber slides (Sigma-Aldrich) were treated for 2 hours at 4°C with 10 *μ*g/mL nocodazole (Calbiochem) to depolymerise microtubules. Cells were then washed with cold PBS to remove nocodazole and subsequent microtubule regrowth assessed upon replacement of culture medium at 0, 5, and 15 minutes, at 37°C. Where stipulated, 100 nM CEP-701 was added during the last 30 minutes of nocodazole treatment and in regrowth medium.

To visualise microtubules, cells were permeabilized for 30 seconds in 80 mM Pipes, pH 6.8, 5 mM EGTA, pH 8.0, 1 mM MgCl_2_, and 0.5% Triton X-100, fixed for 10 minutes in the same buffer containing 5% glutaraldehyde, and incubated for 7 minutes in 1% sodium borohydride in PBS. Cells were then stained with antibodies against *α*-tubulin (Santa Cruz) and *γ*-tubulin (Sigma-Aldrich) and washed in PBS prior to incubation with appropriate anti-mouse Texas-red conjugated and anti-rabbit FITC-conjugated secondary antibodies (Jackson Immune Research). Nuclear chromatin was counterstained with DAPI (Vector Labs). IF images were obtained at a constant exposure time to limit overexposure, and *α*-tubulin IF signals radiating from *γ*-tubulin positive centrosomes were measured in two separate concentric circles centred at the centrosome with radii of 1 and 2 *μ*m, with background fluorescence subtracted using circles of corresponding sizes, using IF Jpeg images and ImageJ software [36]. MT regrowth areas and centrosome sizes were quantified by measuring respective *α*- and *γ*-tubulin IF areas calculated from outlined areas, using ImageJ software [36].

### 2.4. Immunoprecipitation and Western Blots

Cells were extracted in lysis buffer (PBS containing 0.5% sodium deoxycholate, 1% NP40, 0.1% SDS, 1 mM sodium orthovanadate, 1 mM PMSF, 1 *μ*g/mL of pepstatin A, and Aprotinin) and protein concentrations calculated by Bradford protein concentration assay (Sigma-Aldrich). Prior to immunoprecipitation, extract aliquots (200–500 *μ*g) were precleared with 1 *μ*g of preimmune IgG (1 hour at 4°C) and 20 *μ*L of Protein A Sepharose (Fast Flow, Sigma), for 20 minutes at 4°C. For immunoprecipitation, 200–500 *μ*g of extract was incubated with antibody at a concentration range of 0.1–1.0 *μ*g/500 *μ*g total protein for 2–16 hours at 4°C. Following incubation, 20 *μ*L of Protein A Sepharose (Fast flow, Sigma-Aldrich) in lysis buffer was added and reactions incubated for 30 minutes at 4°C. Protein Sepharose/IgG conjugates were collected by centrifugation (10,000 ×g for 5 minutes), washed 3 times in lysis buffer, resuspended in SDS-PAGE sample buffer, and subjected to reducing SDS-PAGE/Western blotting. Briefly, proteins were transblotted by electrophoresis onto Hybond C+ nitrocellulose membranes (Amersham Int. UK) and air-dried. Nonspecific binding sites on membranes were blocked by incubation for 2 hours in 5% nonfat milk in TBS prior to incubation with primary antibodies at recommended dilutions for 2–16 hours at 4°C, washed in TBS, and then incubated with secondary HRP-conjugated antibodies diluted in blocking solution. Immunoreactive species were detected by chemiluminescence reaction as directed (Amersham Int., Bedford, UK).

### 2.5. *α*-Tubulin Kinase and Polymerisation Assays

Tubulin polymerisation assays were performed as previously described [25]. Control, TrkAI, and TrkAIII immunoprecipitates were prepared from respective SH-SY5Y transfectants by incubating total cell extracts (400 *μ*g) with 1 *μ*g of anti-TrkA (C-14) antibody for 2 hours at 4°C, followed by incubation with 20 *μ*L of Protein A Sepharose suspension (Fast flow, Sigma-Aldrich). Protein A immunoprecipitates were recovered by centrifugation at 15,000 rpm in a microfuge at 4°C and washed 3 times in RIPA buffer and 2 times in 50 mM Tris-HCl (pH 7.5). Two vials were prepared each containing a 9 : 1 ratio of unlabelled (9 parts) bovine brain *α*/*β*-tubulins and rhodamine-labelled (1 part) bovine brain *α*-tubulin (Cytoskeleton Inc.), resuspended in general tubulin buffer (80 mM Pipes (pH.6.8), 1 mM MgCl_2_, and 1 mM EGTA) containing 1 mM GTP (Sigma-Aldrich). To the first vial, 20 *μ*L of general tubulin buffer containing 2 mM GTP and ATP was added to a final concentration of 100 *μ*M. The second vial received 23 *μ*L of general tubulin buffer containing 2 mM GTP but not ATP. Washed Protein A Sepharose immunoprecipitates were resuspended in either 15 *μ*L of non-labelled tubulin/rhodamine *α*-tubulin (9 : 1) in the presence or absence of ATP and incubated for 1 hour at 37°C. Reaction samples (3–5 *μ*L) were subsequently removed and either (a) mixed with reducing SDS-PAGE sample buffer for Western blotting to examine *α*-tubulin tyrosine phosphorylation or (b) mixed with an equal volume of general tubulin buffer containing 60% v/v glycerol on ice, spread onto glass slides, covered with a glass coverslip, and examined by fluorescent microscopy for the presence of rhodamine-labelled tubulin polymers.

## 3. Results

### 3.1. TrkAIII Promotes MT Nucleation and Assembly at the Centrosome

**Figure 1:**
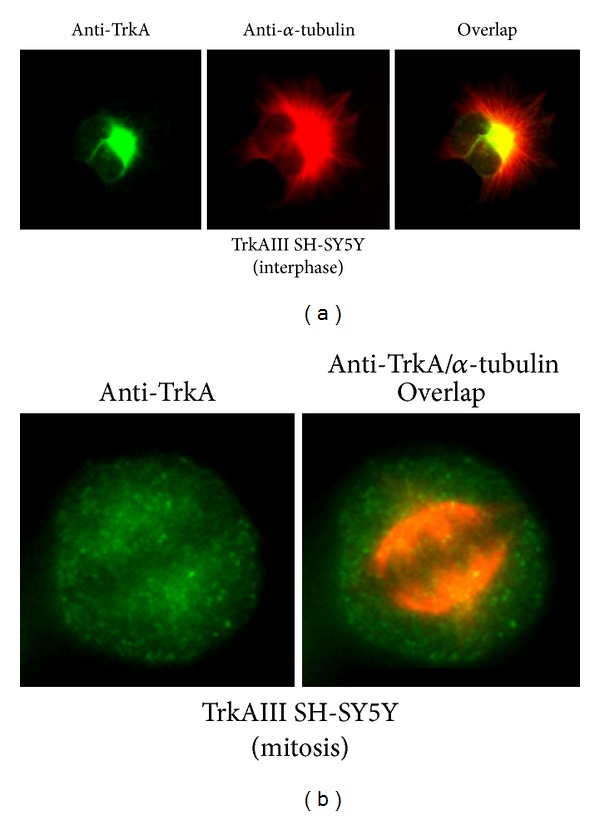
Representative IF images illustrating the typical pattern of TrkAIII expression (green), *α*-tubulin positive MTs (red), and overlap (yellow/orange) in interphase (a) and mitotic (b) TrkAIII SH-SY5Y transfectants.

**Figure 2:**
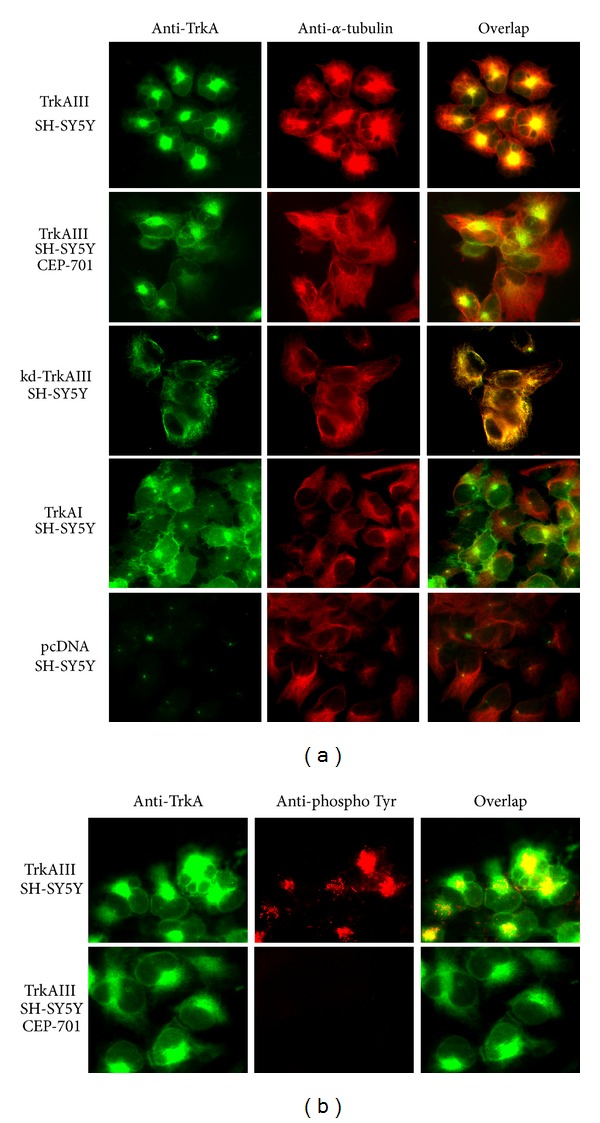
(a) Representative IF images comparing TrkA variant expression (green), *α*-tubulin positive MTs (red), and the overlap (yellow/orange) in untreated and CEP-701-treated (100 ng overnight) TrkAIII SH-SY5Y transfectants and in untreated kd-TrkAIII, TrkAI, and control pcDNA SH-SY5Y transfectants. (b) Representative IF images comparing the pattern of TrkAIII expression (green), TrkAIII-associated tyrosine phosphorylation (red), and overlap (yellow/orange) in untreated and CEP-701-treated (100 nM, overnight) TrkAIII SH-SY5Y transfectants.

Indirect IF detected intense arrays of *α*-tubulin positive MTs in TrkAIII SH-SY5Y transfectants, radiating outwards from a perinuclear focal point consistent with a centrosomal MTOC origin during interphase. This pattern of MT assembly exhibited marked overlap with intracellular TrkAIII, which was concentrated to the pericentrosomal region but was not detected throughout the cytoplasm or at the cell periphery (Figure 1(a)). Significant overlap between TrkAIII and *α*-tubulin positive MTs was not detected during mitosis (Figure 1(b)). This pattern of *α*-tubulin positive MTs was markedly altered following overnight treatment with 100 nM CEP-701, which was also associated with a reduction in TrkAIII overlap with *α*-tubulin (Figure 2(a)). 

In contrast to TrkAIII transfectants, neither kinase dead kd-TrkAIII, TrkAI, nor control SH-SY5Y transfectants exhibited this pattern of *α*-tubulin positive MT assembly, with all three cell lines characterised by less intense, more diffuse cytoplasmic MT distribution, which was less focalised at the centrosome (Figure 2(a)). CEP-701 inhibited TrkA-associated tyrosine phosphorylation, used as a surrogate for TrkAIII tyrosine kinase activity, at the dose employed in this study (Figures 2(b) and 4(b)).

### 3.2. MT Regrowth Assays

**Figure 3:**
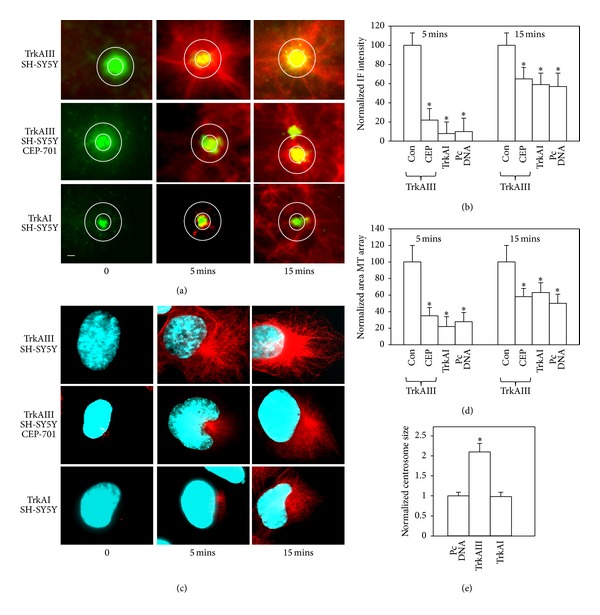
Representative IF images and histograms of MT regrowth assays, demonstrating (a) indirect IF changes in *α*-tubulin positive MT regrowth (red) from the *γ*-tubulin positive centrosome (green) in untreated and CEP-701-treated (100 nM) TrkAIII SH-SY5Y transfectants and in untreated TrkAI SH-SY5Y transfectants at 0, 5, and 15 minutes after nocodazole washout. (b) Histogram demonstrating the differences in mean (±S.E.) *α*-tubulin IF intensity in untreated and CEP-701-treated (100 nM for 1 hour) TrkAIII SH-SY5Y transfectants and in untreated TrkAI and control pcDNA SH-SY5Y transfectants at 0, 5, and 15 minutes after nocodazole washout, normalised with respect to untreated TrkAIII transfectants (arbitrary value 100%; *n* = 50 per group; * = statistical significance). (c) Indirect IF demonstrating differences in *α*-tubulin positive MT regrowth area (red) in untreated and CEP-701-treated (100 nM) TrkAIII SH-SY5Y transfectants and in untreated TrkAI SH-SY5Y transfectants at 0, 5, and 15 minutes after nocodazole washout. (d) Histogram demonstrating differences in *α*-tubulin positive MT regrowth area in untreated and CEP-701-treated (100 nM) TrkAIII SH-SY5Y transfectants and in untreated TrkAI and control pcDNA SH-SY5Y transfectants at 0, 5, and 15 minutes after nocodazole washout, normalised with respect to untreated TrkAIII transfectants (arbitrary value of 100%; *n* = 50 per group; * = statistical significance). (e) Histogram demonstrating the differences in *γ*-tubulin positive centrosome size in untreated control, TrkAI, and TrkAIII SH-SY5Y transfectants, normalised with respect to untreated control transfectants given the arbitrary value of 1 (*n* = 50 per group; * = statistical significance).

The centrosomal origin of *α*-tubulin positive MT arrays in TrkAIII transfectants was confirmed by contemporary *α*-tubulin and *γ*-tubulin IF (Figure 3(a)).

In MT regrowth assays, TrkAIII transfectants exhibited significantly more rapid MT regrowth at 5 and 15 minutes following nocodazole washout, in terms of both recovery of pericentrosomal *α*-tubulin IF intensity initiating at the *γ*-tubulin positive centrosome (Figures 3(a) and 3(b)) and total MT regrowth area (Figures 3(c) and 3(d)), when compared to untreated control and TrkAI transfectants and CEP-701-treated TrkAIII transfectants (100 nM, 30 minutes before treatment and for the duration of assay). When compared to untreated TrkAIII transfectants normalised to an arbitrary value of 100%, CEP-701-treated TrkAIII transfectants exhibited a significant 78% reduction (*P* ≤ 0.002, *n* = 50) in pericentrosomal *α*-tubulin IF intensity, 65% reduction (*P* ≤ 0.003, *n* = 50) in total MT regrowth area at 5 minutes, a significant 40% reduction (*P* ≤ 0.027, *n* = 50) in pericentrosomal *α*-tubulin IF intensity, and 41% reduction in total MT regrowth area (*P* ≤ 0.032, *n* = 50) at 15 minutes, after nocodazole washout (Figures 3(b) and 3(d)); TrkAI transfectants exhibited a significant 82% reduction (*P* ≤ 0.0001, *n* = 50) in pericentrosomal *α*-tubulin IF intensity, 78% reduction (*P* ≤ 0.0001, *n* = 50) in total MT regrowth area at 5 minutes, a significant 41% reduction (*P* ≤ 0.026, *n* = 50) in pericentrosomal *α*-tubulin IF intensity, and 37% reduction in total MT regrowth area (*P* ≤ 0.038, *n* = 50) at 15 minutes, after nocodazole washout (Figures 3(b) and 3(d)); and control transfectants exhibited significant 78% reduction (*P* ≤ 0.006, *n* = 50) in pericentrosomal *α*-tubulin IF intensity, 72% reduction (*P* ≤ 0.0001, *n* = 50) in total MT regrowth area at 5 minutes, significant 43% reduction (*P* ≤ 0.027, *n* = 50) in pericentrosomal *α*-tubulin IF intensity, and 50% reduction in total MT regrowth area (*P* ≤ 0.005, *n* = 50) at 15 minutes, after nocodazole washout (Figures 3(b) and 3(d)).

Measurement of the area of *γ*-tubulin IF, as an estimate of relative centrosome size, revealed that centrosomes in TrkAIII transfectants were significantly (*P* ≤ 0.001, *n* = 50) 2.1 ± 0.14-fold larger than centrosomes in control transfectants (normalised to an arbitrary value of 1.0 ± 0.07, *n* = 50) and TrkAI transfectants, which were not significantly different in size to control centrosomes (0.98 ± 0.12, *n* = 50, *P* ≤ 0.89 NS) (Figure 3(d)).

### 3.3. TrkAIII Binds and Phosphorylates *α*-Tubulin and Promotes MT Assembly In Vitro

**Figure 4:**
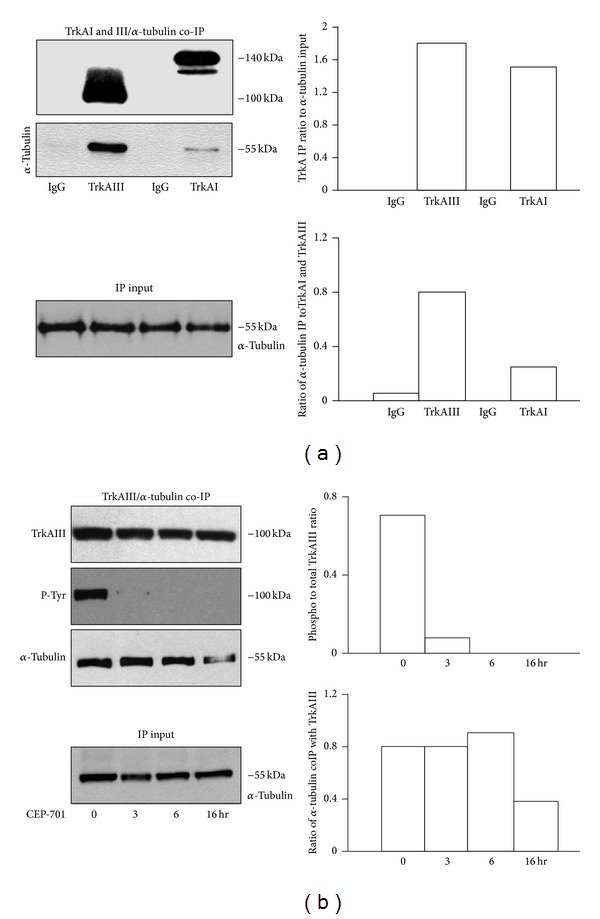
(a) IP/Western blots demonstrating differences in *α*-tubulin levels pulled down by TrkAIII and TrkAI immunoprecipitates from respective SH-SY5Y transfectants, plus histograms displaying densitometric analysis of the adjacent blots, demonstrating the presence of similar levels of TrkAI and TrkAIII immunoprecipitates, normalised to input *α*-tubulin levels, plus the difference in *α*-tubulin levels pulled down as a densitometric ratio to TrkAI and TrkAIII. (b) IP/Western blots demonstrating the effect of CEP-701 (100 nM for 0–16 hours) on TrkAIII tyrosine phosphorylation (P-Tyr) and *α*-tubulin levels pulled down in coimmunoprecipitation assays, plus histograms displaying densitometric analysis of the adjacent blots, demonstrating CEP-701-induced loss of TrkAIII tyrosine phosphorylation from 3 hr onwards, associated with reduced *α*-tubulin binding at 16 hours only.

In coimmunoprecipitation assays, TrkAIII pulled down a greater quantity of *α*-tubulin when compared to an equivalent amount of immunoprecipitated TrkAI, normalised to *α*-tubulin levels in input extracts (Figure 4(a)). CEP-701 (100 nM) inhibited TrkAIII tyrosine phosphorylation at times from 3 hours onward (Figure 4(b)) and caused a moderate reduction in *α*-tubulin/TrkAIII coimmunoprecipitation at 16 hours but not before (Figure 4(b)).

**Figure 5:**
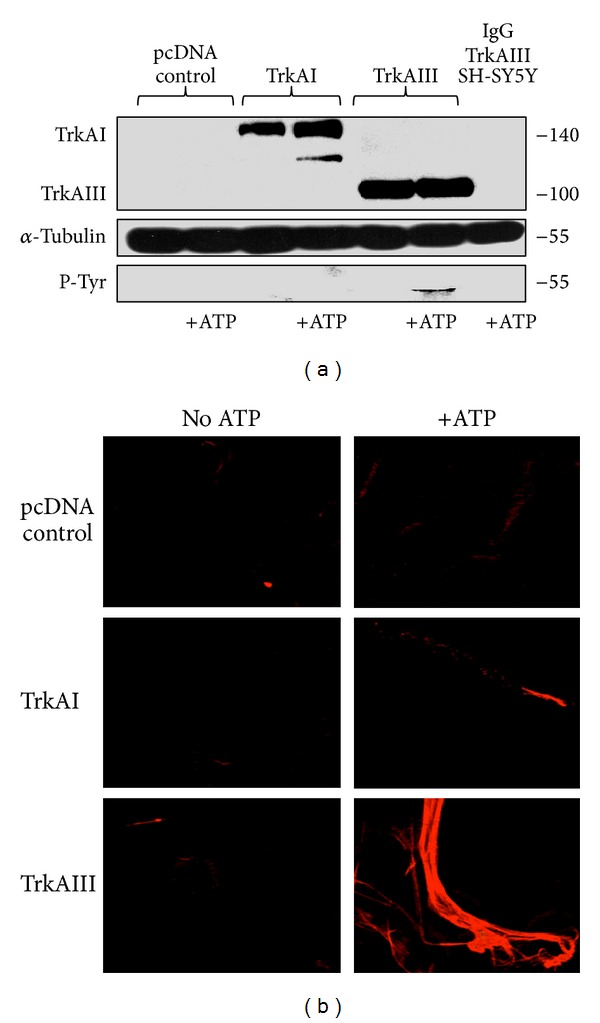
(a) Western blots demonstrating the relative levels of TrkAI, TrkAIII, and total tyrosine phosphorylated *α*-tubulins in a representative *α*-tubulin phosphorylation assay; (b) IF images demonstrating the difference in tubulin polymerisation induced by TrkAIII but not control or TrkAI immunoprecipitates in a representative tubulin polymerisation assay, in the presence but not in the absence of ATP.

In tubulin polymerisation assays, TrkAIII but not an equivalent amount of TrkAI immunoprecipitate induced low but detectable tyrosine phosphorylation of exogenous *α*-tubulin (Figure 5(a)) and promoted the polymerisation of tubulin *in vitro*, in the presence but not in the absence of ATP (Figure 5(b)).

### 3.4. TrkAIII Promotes Nuclear Lobulation

**Figure 6:**
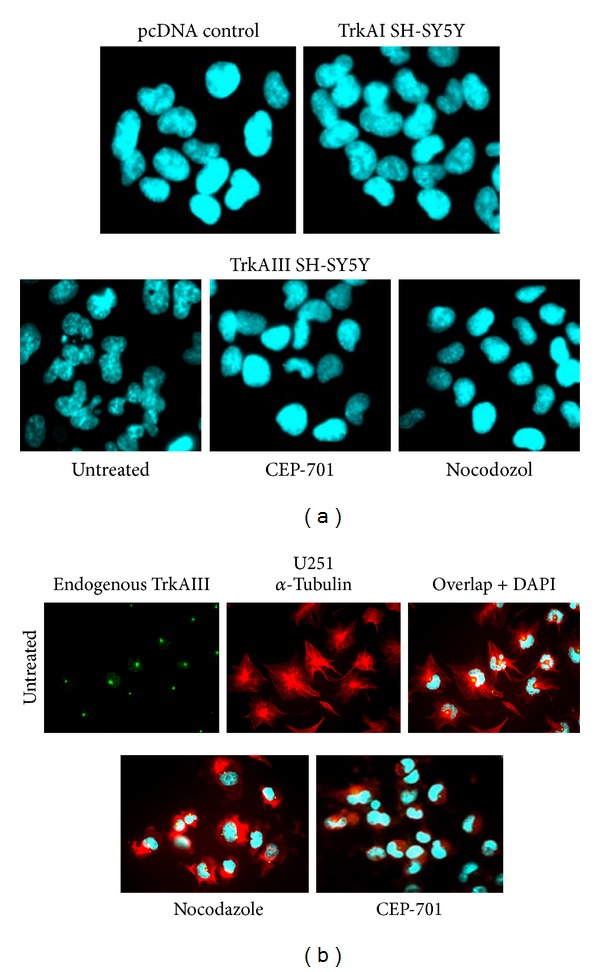
(a) Representative IF images demonstrating the difference in the regular oval nuclear morphology exhibited by pcDNA control and TrkAI SH-SY5Y transfectants, compared to the lobulated nuclei in TrkAIII SH-SY5Y transfectants, plus the inhibition of nuclear lobulation in TrkAIII SH-Sy5Y transfectants following incubation with CEP-701 (100 nM for 16 hours) and nocodazole (10 *μ*g/mL for 2 hours). (b) Representative IF images demonstrating the intracellular distribution of endogenous TrkAIII (green), *α*-tubulin positive MTs (red), TrkAIII/*α*-tubulin overlap (yellow/orange), and DAPI-stained nuclei (blue) in human U251 cells, plus IF images demonstrating the capacity of nocodazole (10 *μ*g/mL for 2 hours) and CEP-701 (100 nM for 16 hours) to inhibit nuclear lobulation in U251 cells.

TrkAIII but not TrkAI or control SH-SY5Y transfectants exhibited a highly lobular nuclear morphology (Figure 6(a)). Nuclear lobulation was detected during interphase, lost during prophase in association with MT depolymerization (not shown), and inhibited by both CEP-701 (100 nM for 16 hours) and nocodazole (10 *μ*g/mL for 2 hours) (Figure 6(a)). Human U251 glioblastoma cells, which express endogenous TrkAIII that localises to the centrosome [4], also exhibited a highly lobulated nuclear morphology (Figure 6(b)) that was inhibited by both CEP-701 (100 nM for 16 hours) and nocodazole (10 *μ*g/mL for 2 hours), in association with the rearrangement of *α*-tubulin positive MTs (Figure 6(b)).

## 4. Discussion

In this study, we identify a novel role for TrkAIII in promoting the nucleation and assembly of MTs at the centrosome in human SH-SY5Y NB cells. We propose that this function depends upon spontaneous intracellular TrkAIII activation and the capacity of TrkAIII to bind *α*- and *γ*-tubulins, to localise to the centrosome, and to promote tubulin polymerisation, contributing to MT involvement in the promotion and maintenance of a proliferating, undifferentiated, and anaplastic NB cell phenotype by restricting and augmenting MT nucleation and assembly at the centrosomal MTOC.

The striking difference in *α*-tubulin positive MT assembly exhibited by TrkAIII but not control or TrkAI transfectants, characterised by relatively short intense MT arrays radiating outwards from the centrosome, suggests a role for TrkAIII tyrosine kinase activity in MT assembly. This possibility is supported by the observations that (a) MT assembly and nucleation at the centrosome in TrkAIII transfectants were reduced by CEP-701 at TrkAIII inhibitory concentrations; (b) kd-TrkAIII SH-SY5Y transfectants did not exhibit this pattern of MT nucleation and assembly; and (c) TrkAIII SH-SY5Y transfectants exhibited significantly more rapid MT regrowth from the centrosome, when compared to control, TrkAI, and CEP-701-treated TrkAIII transfectants.

The formation of relatively short MT arrays in TrkAIII transfectants, radiating outward from the *γ*-tubulin positive centrosome, bears close similarity to MT assembly in undifferentiated cells [11–15], suggesting that TrkAIII promotion and maintenance of an undifferentiated NB phenotype may depend, at least in part, upon the restriction and augmentation of MT nucleation and assembly at the centrosomal MTOC. This differs from MT reorganisation, nucleation, and assembly associated with neuronal differentiation induced either by neurotrophin-activated cell surface TrkA or cytoplasmic Fes, which is characterised by the formation of long MT processes required for neuritogenesis, growth cone formation, and axon genesis, which nucleate also from noncentrosomal MTOCs and are reorganised at the cell periphery [11, 21, 25, 29–32]. This difference may be explained by tyrosine kinase localisation, since TrkAIII exhibits spontaneous intracellular activation in the pericentrosomal region and at the centrosome [4], whereas neurotrophins activate fully spliced TrkA at the cell surface [1, 29–31], and c-Fes is activated throughout the cytoplasm [25]. Furthermore, the centralized location exhibited by activated TrkAIII (this study and [4]) may also help to explain the pericentrosomal overlap exhibited by TrkAIII and MTs, which did not extend throughout the cytoplasm, as reported for activated Fes [25], nor to the cell periphery, as reported for the interaction between MTs and TrkA within lipid rafts [21].

TrkAIII promotion of MT nucleation and assembly at the centrosome bears close similarity to the influence of c-Src upon MT nucleation and assembly, which has been reported to depend upon the recruitment of *γ*-tubulin ring structures to the centrosome [23, 24]. TrkAIII binds *γ*-tubulin [4] and may also interact with c-Src [37], suggesting that TrkAIII could also import *γ*-tubulin ring structures to the centrosome either directly or indirectly. In support of this, *γ*-tubulin positive centrosomes in TrkAIII transfectants were significantly larger than centrosomes in control or TrkAI transfectants. We are currently investigating potential c-Src involvement in this observation. Alternatively, TrkAIII may bind and not import *γ*-tubulin to the centrosome, limiting its potential influence to MT assembly after nucleation.

Spontaneous TrkAIII activation is restricted to interphase in SH-SY5Y cells [3–5], indicating that TrkAIII influence upon MT assembly may also be restricted to interphase. This is supported by the observation that TrkAIII expression did not inhibit proliferation, as occurs with terminal differentiation, indicating that MT remodelling required for cell cycle progression was not compromised [11, 14] and also explaining why we did not detect TrkAIII association with the mitotic spindle, as previously reported for tyrosine phosphorylated TrkA [32].

The capacity of TrkAIII to bind *α*-tubulin adds to its capacity to bind *γ*-tubulin [4]. TrkAIII tyrosine kinase involvement in *α*-tubulin binding is supported by the relatively low level of *α*-tubulin binding exhibited by TrkAI and the modest reduction in *α*-tubulin binding by TrkAIII following overnight treatment with CEP-701 and is corroborated by reports that phosphorylated TrkA colocalises with *α*-tubulin [32], activated TrkA interacts with and modifies *α*-tubulin [33], neurotrophin activated TrkA recruits and reorganises MTs in lipid rafts during neuron differentiation [21, 29, 30], and retrograde transport of activated TrkA is mediated by dynein/MT interaction [34]. Whether TrkAIII interacts directly with *α*-tubulin or indirectly via dynein [34], c-Src [23, 37], and/or perhaps FRS-3 [38] remains to be elucidated. However, the fact that TrkAIII contemporarily binds *α* and *γ*-tubulin (this study and [4]) suggests that TrkAIII may independently recruit *α* and *γ*-tubulin to the centrosome for MT nucleation and assembly.

TrkAIII promotion of MT assembly *in vivo*, exemplified by the exaggerated pattern of MT assembly in TrkAIII transfectants and confirmed in MT regrowth assays, was further supported in tubulin polymerisation assays* in vitro*, which unveiled the capacity of TrkAIII but not TrkAI or control immunoprecipitates to induce low but detectable tyrosine phosphorylation of exogenous *α*-tubulin and to promote tubulin polymerisation. Whether this results directly from TrkAIII tyrosine kinase activity or TrkAIII-associated tyrosine kinases, such as c-Src [23, 35], remains to be elucidated. However, it is clear that spontaneously active TrkAIII acts in a manner analogous to neurotrophin-activated cell surface TrkA in its capacity to reorganise and promote MT assembly *in vivo *but does so at the centrosome rather than cell periphery, resulting in the promotion and maintenance of a proliferating, undifferentiated NB cell phenotype rather than inducing neuronal differentiation, which results from cell surface TrkA activation (this study [1, 8–10]).

The undifferentiated phenotype exhibited by TrkAIII SH-SY5Y transfectants was also accompanied by a lobular nuclear morphology. TrkAIII tyrosine kinase and MT involvement in nuclear lobulation was confirmed using CEP-701 and nocodazole, both of which inhibited nuclear lobulation. Human U251 glioblastoma cells, which express endogenous TrkAIII that localises to the centrosome [4], also exhibited a highly lobular nuclear morphology similar to that of TrkAIII SH-SY5Y transfectants, associated with intense MT arrays, radiating outward from the centrosome and overlapping pericentrosomal and centrosomal endogenous TrkAIII. As for TrkAIII SH-SY5Y transfectants, nuclear lobulation in U251 cells was inhibited by both CEP-701 and nocodazole, confirming close similarity between exogenous and endogenous TrkAIII.

## 5. Conclusions

In conclusion, we propose that spontaneous intracellular pericentrosomal TrkAIII activation contributes to MT involvement in the promotion and maintenance of a proliferating, undifferentiated, and anaplastic NB cell phenotype by restricting and augmenting MT nucleation and assembly to the centrosomal MTOC. This function depends upon TrkAIII capacity to bind *α*- and *γ*-tubulin, to localise to the centrosome, and to promote tubulin polymerisation.
